# Eyetracking-enhanced VEP for nystagmus

**DOI:** 10.1038/s41598-023-50367-y

**Published:** 2023-12-20

**Authors:** Matt J. Dunn, Perry Carter, Jay Self, Helena Lee, Fatima Shawkat

**Affiliations:** 1 School of Optometry and Vision Sciences, Cardiff University, Maindy Road, Cardiff, CF24 4HQ Wales, UK; 2 Southampton Eye Unit, University Hospital Southampton NHS Foundation Trust, Southampton, UK; 3 Clinical and Experimental Sciences, Faculty of Medicine, University of Southampton, Southampton, UK

**Keywords:** Ocular motility disorders, Biomedical engineering

## Abstract

Visual evoked potentials (VEPs) are an important prognostic indicator of visual ability in patients with nystagmus. However, VEP testing requires stable fixation, which is impossible with nystagmus. Fixation instability reduces VEP amplitude, and VEP reliability is therefore low in this important patient group. We investigated whether VEP amplitude can be increased using an eye tracker by triggering acquisition only during slow periods of the waveform. Data were collected from 10 individuals with early-onset nystagmus. VEP was obtained under continuous (standard) acquisition, or triggered during periods of low eye velocity, as detected by an eye tracker. VEP amplitude was compared using Bonferroni corrected paired samples t-tests. VEP amplitude is significantly increased when triggered during low eye velocity (95% CI 1.42–6.83 µV, t(15) = 3.25, p = 0.0053). This study provides proof-of-concept that VEP amplitude (and therefore prognostic reliability) can be increased in patients with early onset nystagmus by connecting an eye tracker and triggering acquisition during periods of lower eye velocity.

## Introduction

Infantile nystagmus (IN) is a condition characterized by involuntary rhythmic movements of the eyes. The condition typically appears during infancy and may worsen during periods of stress or fatigue^1,2^. IN is often associated with an underlying pathology of the visual system, but sometimes appears with no detectable comorbidity (idiopathic IN). Individuals with IN typically have reduced visual function^3^.

Visual evoked potentials (VEP) identify and quantify disorders of the visual system and assess the function of the afferent pathway from retina to cortex. Pattern VEP testing, using different sizes of black and white checkerboard patterns that either alternate (pattern reversal) or appear/disappear (onset/offset VEPs), is used to measure visual sensitivity. These tests form an important adjunct to the basic clinical examination of patients and in particular, infants presenting with nystagmus^4^.

While electroretinography (ERG) is used to identify retinal disorders associated with nystagmus, VEPs are important in determining the extent of macular involvement and gaining insight into visual potential. Furthermore, VEPs are used to assess the integrity of the post-retinal pathway as some neurological disorders, such as optic nerve hypoplasia and gliomas, and conditions associated with chiasmal anomalies such as albinism and achiasma, often present with nystagmus.

Individuals with IN have degraded pattern VEPs. Nystagmus eye movements per se contribute towards this VEP degradation^5^, and this confounding factor can affect interpretation of results. In adults with idiopathic IN, onset-offset VEPs produce higher amplitude signals that are more readily distinguished from noise, than pattern reversal VEPs^6^.

A retrospective analysis of pattern reversal VEPs from 26 children with IN found that by selectively averaging VEP reversals with similar amplitude and phase across Fourier-domain frequencies, signal amplitude increased by 270–420%, and signal-to-noise ratio increased by 188–530%. The authors concluded this was because they were rejecting reversals falling outside of foveation periods^7^. Another study by the same group found higher VEP amplitude for signals coinciding with periods of well-defined foveations^5^.

The present study investigated whether VEP amplitude can be increased in early-onset nystagmus by connecting an eye tracker and triggering acquisition only when eye velocity is low.

## Results

**Figure 1:**
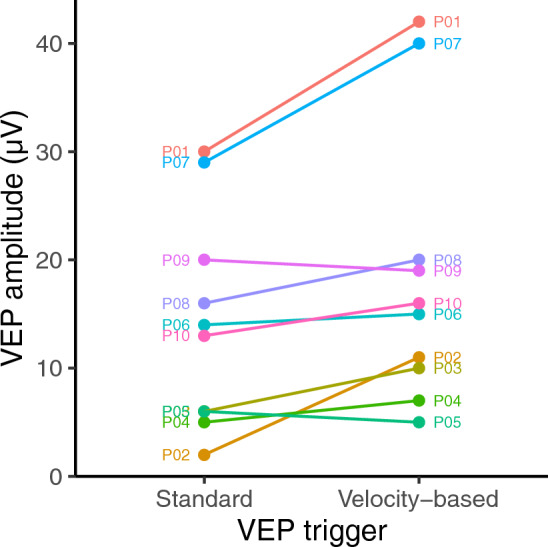
Amplitudes obtained for onset-offset VEPs using both standard and velocity-based triggering.

**Figure 2:**
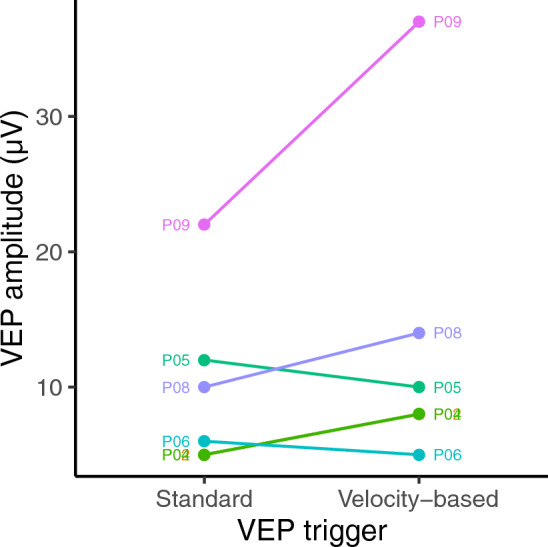
Amplitudes obtained for pattern reversal VEPs using both standard and velocity-based triggering. Note that P02 and P04 are coincident.

Figures 1 and 2 show VEP amplitudes obtained using onset-offset and pattern reversal VEPs respectively. A paired samples t-test indicated no significant difference in the VEP amplitudes obtained for onset-offset vs pattern reversals (95% CI − 5.03 to 4.69 µV, t(11) =  − 0.075, p = 0.94). Therefore, onset-offset and pattern reversal VEPs were analysed together. A paired samples t-test indicated that VEP amplitude was significantly higher with velocity triggering than under continuous (standard) triggering (95% CI 1.42–6.83 µV, t(15) = 3.25, p = 0.0053).

Velocity-triggering resulted in a mean of 121 VEP epochs per recording (± 25 standard error), whereas continuous acquisition resulted in 134 epochs per recording (± 12 standard error). A paired-samples t-test found no significant difference in the number of epochs obtained between acquisition methods (95% CI − 67 to 78, t(10) = 0.16, p = 0.87).

A linear regression model was fitted to examine the relationship between trigger velocity threshold and the proportional increase in VEP amplitude using velocity triggering. This was not significant (p = 0.16), indicating that improvements in VEP amplitude do not depend on the velocity trigger threshold used across participants.

**Figure 3:**
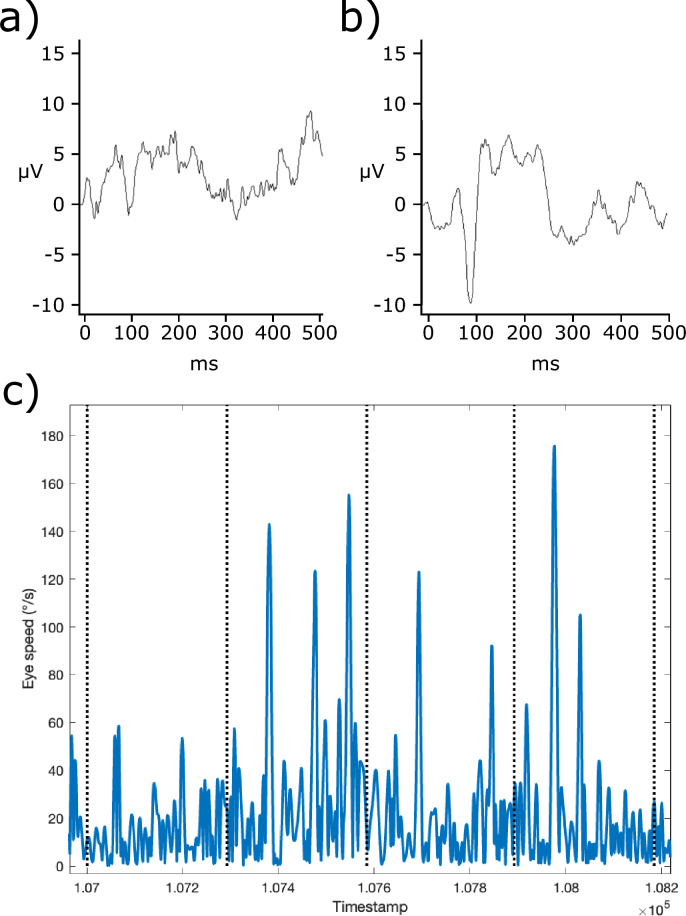
Example data (from P10) showing averaged onset-offset VEP waveform under (**a**) continuous triggering and (**b**) velocity triggering, and (**c**) eye speed trace with vertical dashed lines indicating trigger times. Note that VEP acquisition occurs ~ 24 ms after trigger.

Figure 3 shows an example averaged VEP waveform and a portion of the eye speed trace which provided the triggers for VEP acquisition.

## Discussion

This study demonstrates that VEP amplitude can be increased in early onset nystagmus by triggering VEPs when the eyes move slowly. This method has the potential to improve prognostic reliability.

Previous studies investigated periods of low eye velocity during continuous VEP acquisition^5,7^, whereas our method triggers VEP acquisition only when the eyes are moving slowly. We suggest two possible reasons that VEP amplitude is higher when the eyes move slowly. One is that reduced motion blur when the eyes are slowed improves sensitivity, but this is at odds with evidence that motion blur does not limit visual acuity in adults with IN^8^ (only one participant in the present study was within the critical period of visual development). Another possibility is that visual sensitivity is reduced during non-foveating periods of the nystagmus waveform, but the ‘visual sampling’ hypothesis is also contested^9^.

IN waveforms typically include regular foveation periods during which eye velocity is slowest. The ‘velocity trigger’ used in our method is therefore likely best suited to IN waveforms with established foveations periods. Four of our participants with IN (P02, P03, P07 and P10) exhibited well-defined foveation periods; further research with a larger sample size could determine the extent to which a patient’s foveation quality predicts the benefits of using our method. Further investigation in patients with late-onset nystagmus may also be warranted.

In conclusion, we provide proof-of-concept that triggering VEP acquisition during slower portions of the nystagmus waveform increases signal amplitude (and therefore reliability) in patients with early onset nystagmus.

## Methods

The study was conducted at the Eye Unit’s Electrodiagnostic Department at University Hospital Southampton. The data collected in the study were approved by the University Hospital Southampton Foundation Trust Governance Committee. The investigation was carried out in accordance with the Declaration of Helsinki; informed consent was obtained from the participants after explanation of the nature and possible consequences of the study. Study participants were identified through a regional nystagmus clinic. All participants had a full history taken and underwent orthoptic assessment, ophthalmic examination of anterior and posterior segments using age-appropriate equipment, detailed nystagmus examination, and optical coherence tomography (OCT) of their maculae using age-appropriate equipment, according to a standard nystagmus evaluation workflow^4,10^. Ten participants with nystagmus took part in the study; Table 1 provides clinical details, including nystagmus subtype. For the purposes of analysis, participants whose nystagmus developed in infancy were classified as ‘early onset’.

**Table 1:** Clinical participant data under binocular viewing.

Participant	Age/sex	Diagnostic group	Clinical details	Clinical VA (logMAR)	Primary position waveform			
					Type	Primary axis	Amplitude (°)	Frequency (Hz)
P01	35/F	IN Possible retinal disorder but clinical genomic testing inconclusive	High myopia. OCT showing macular photoreceptor disruption. Genomic testing inconclusive	R: 1.30 L: 1.20	P	Horiz	–	2.6
P02	41/M	IN Clinical Albinism diagnosis but clinical genomic testing inconclusive	Grade 3 foveal hypoplasia. Normal ERG and crossed asymmetry on VEP testing. Genomic testing inconclusive	R: 0.30 L: 0.18	P_FS_	Horiz	1.1	3.0
P03	30/M	IN Congenital stationary night blindness	Myopia. Electronegative ERG. Genomic testing conclusive for pathogenic *CACNA1F* variant	R: 1.00 L: 0.80	PP_FS_	Horiz	–	–
P04	30/F	IN Clinical Albinism diagnosis but clinical genomic testing inconclusive	Grade 3 foveal hypoplasia. Normal ERG and crossed asymmetry on VEP testing. Genomic testing inconclusive	R: 0.30 L: 0.40	Downbeat	Vert	–	–
P05	14/M	IN Undiagnosed genetic disorder	Normal OCT macula. Normal ERG and crossed asymmetry on VEP. Dysmorphic features. Hearing deficit with preauricular skin tags. Cleft lip and palate surgery. Ventricular septal defect surgery. Developmental delay	R: 0.40 L: 0.78	Irregular	Vert	–	3.0
P06	16/M	IN Born premature at 29 weeks gestational age. Cerebral palsy and hydrocephalus with no other cause for nystagmus found	Normal ERG, VEP and OCT. Abnormal MRI: ventriculomegaly; white matter atrophy; small cerebellum, midbrain, pons and medulla; small optic nerves and chiasm. Genomic testing inconclusive	R: 0.30 L: 1.00	Downbeat/irregular	Vert	11.5	3.1
P07	6/M	IN Albinism	Grade 3 foveal hypoplasia on OCT. Crossed asymmetry on VEP. Genomic testing confirming a pathogenic OCA1 genotype	R: 0.30 L: 0.20	JL_EF_	Horiz	–	–
P08	39/F	IN Albinism	Normal ERG, VEP and OCT. Genomic testing inconclusive	–	JL	Horiz	–	4.0
P09	13/F	FMNS	Refractive error. Strabismus. Amblyopia. Normal ERG, VEP and OCT	R: 0.10 L: 0.40	latent nystagmus	Horiz	0.6	2.8
P10	40/M	IN Idiopathic	Normal ERG, VEP and OCT. Genomic testing inconclusive	R: 0.50 L: 0.42	JL_EF_	Horiz	10.5	3.3

Nystagmus waveform data from eye tracker recordings. Missing values either not provided by clinician or insufficient continuous calibrated eye tracker data available to provide reliable metric. Waveform data reported for participant with FMNS given under monocular viewing.

*FMNS* fusion maldevelopment nystagmus syndrome, *JL* pure jerk left, *JL(*_*EF*_*)* jerk left (with extended foveation), *MRI* magnetic resonance imaging, *P* pure pendular, *(P)P*_*FS*_ (pseudo) pendular with foveating saccades.

Clinical visual acuity was measured either by the orthoptist or referring clinician and converted to logMAR where appropriate. Participants took as long as they wished to view the chart and were encouraged to use their null zone, if present. VEPs were then acquired using an Espion 300 Desktop System (Diagnosis LLC, Cambridge, UK) running Espion software version 6.64.14, and VEPs were recorded following ISCEV guidelines for pattern reversal and onset-offset VEPs. Recordings were made with silver/silver chloride electrodes adhered to the skin using 10/20 conductive paste after preparation with SkinPure gel (Nihon Kohden, Tokyo, Japan) to ensure impedances were equal and < 5 kΩ. Electrode placement followed the Queen Square protocol with active electrodes at O1, Oz, O2, and reference and earth placed on the forehead. Recording settings were as follows: 1000 Hz sample rate, 1 Hz low frequency filter and 100 Hz high frequency filter. Room lights were off. VEP acquisition was triggered externally via a LabJack U6 (LabJack, Lakewood, CO, USA) under two conditions (‘continuous’ and ‘velocity triggered’). For each condition, both *onset-offset* and *pattern reversal* VEPs were acquired (six participants undertook *pattern reversal* VEPs, and all 10 participants undertook *onset-offset* VEPs) using a 50′ chequerboard pattern at a viewing distance of 1 m on a 602 × 341 mm monitor (GEST270HB-OD01, ESTECOM, Gunpo, South Korea) and 1600 × 900 pixel resolution, 60 Hz refresh rate and 200 cd/m^2^ maximum luminance. The head was stabilized by a chin rest. Horizontal and vertical eye position were monitored at 500 Hz by an EyeLink 1000 video-based eye tracker (SR Research, Ottawa, ON, Canada; firmware version 5.15) using a Desktop Mount. The left eye was tracked unless there was a marked difference in interocular visual acuity, in which case the eye with the better vision was tracked. Eye tracker calibration, data processing and segmentation of nystagmus waveforms (as reported in Table 1), were achieved using the method described by Dunn et al.^11^. Stimulus presentation was controlled using MATLAB (The MathWorks, Inc., Natick, MA), using Psychophysics Toolbox extensions^12–14^. A 0.25° red circular fixation target was presented in the straight-ahead position. For *onset-offset* VEPs, the pattern was shown for 250 ms and disappeared for a minimum duration of 295 ms; for *pattern reversal* VEPs, reversals occurred at minimum intervals of 333 ms. In the ‘continuous’ condition, VEPs were acquired back-to-back at regular intervals (according to the minimum durations above). In the ‘velocity triggered’ condition, VEP acquisition (either an onset-offset pair or pattern reversal, as appropriate) took place whenever eye velocity fell below a threshold, determined per-participant (see below). Eye velocity was calculated live by differentiation of gaze position over the preceding 10 ms, using a 5 ms averaging window. VEP acquisition was triggered when eye velocity exceeded threshold, with acquisition frequency limited to the minimum intervals specified above. Since nystagmus intensity varies across individuals (and in some cases *within* an individual), VEP trigger threshold was empirically determined at the start of each recording by adjustment by the experimenter until high enough to trigger, but not so high as to trigger continuously (thresholds ranged from 0.5 to 26°/s [mean 7.8 ± 1.5°/s standard error]). Using a photodiode, the total latency of the video hardware was measured as ~ 24 ms; the total latency between the eye velocity trigger and VEP acquisition was adjusted to also be ~ 24 ms (ensuring that stimulus onset coincided with VEP acquisition, both of which occurred ~ 24 ms after the appropriate eye velocity was detected). Using the R Environment for Statistical Computing^15^, a paired-samples t-test was conducted to compare VEP amplitude under each trigger condition (velocity triggered vs continuous).

## Data Availability

The data presented in this article is available at 10.5255/UKDA-SN-856581.
